# Traumatic Lens Dislocation in an Eye with Anterior Megalophthalmos

**DOI:** 10.1155/2022/6366949

**Published:** 2022-05-09

**Authors:** Huda AlGhadeer

**Affiliations:** King Khaled Eye Specialist Hospital, Riyadh, Saudi Arabia

## Abstract

Anterior megalophthalmos is a rare, bilateral, nonprogressive, hereditary, congenital disorder characterized by the enlargement of all anterior segment structures of the eye, with megalocornea, iris atrophy, and zonular abnormalities. We report a case of an 8-year-old male who presented to the emergency department with a history of visual loss after a blunt ocular trauma to the left eye. The patient presented with markedly enlarged corneas and deepened anterior chambers bilaterally. Best-corrected visual acuity (BCVA) was hand motion in the left eye. An additional examination revealed multiple anterior segment abnormalities, leading to the diagnosis of megalophthalmos and lens dislocation in the anterior chamber. The patient underwent a lensectomy and anterior vitrectomy in the left eye. At six months postoperatively, the BCVA was 20/200 in the left eye. Lens dislocation in patients with megalocornea is rare. Cataract surgery in these patients requires attention to the zonular abnormalities and lens enlargement, resulting in increased rates of intraoperative and postoperative complications. Ophthalmologists should be able to diagnose this rare disorder and manage the associations and complications.

## 1. Introduction

In 1914, Wright first described anterior megalophthalmos (AM) [1]. AM is a rare condition involving congenital stationary enlargement of the anterior segment of the eye [1]. It is characterized by nonprogressive, symmetric, bilateral enlargement of the horizontal diameter of the cornea ≥ 13 mm (megalocornea), deep anterior chamber (AC), iris hypoplasia, enlarged ciliary body ring, and the absence of features suggestive of congenital glaucoma [2]. Keratodysgenesis and/or iridogoniodysgenesis likely cause AM [3]. We describe a rare case of lens dislocation in an eye with anterior megalophthalmos following ocular trauma.

### 1.1. Patient Consent Statement

A written consent was obtained from the legal guardian of the patient to publish the details of the case. The medical team takes responsibility for the patient data. The Hospital's Institutional Review Board approved this case report, and it adhered to the tenets of the Declaration of Helsinki.

## 2. Case Report

**Figure 1:**
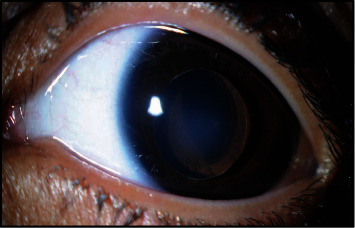
Slit lamp photograph of the left eye showing megalocornea and microspherophakia with lens dislocation in the anterior chamber and ectropion uveae.

An 8-year-old male with megalocornea and microspherophakia presented to the ophthalmic emergency department with redness, pain, and reduced vision five hours after sustaining blunt trauma by a stone to his left eye. The best-corrected visual acuity (BCVA) in the right eye was 20/40 and hand motion in the left eye. Apart from megalocornea and microspherophakia, ocular examination in the right eye was unremarkable. Slit lamp examination of the left eye indicated complete lens dislocation in the anterior chamber with an intact capsule and ectropion uveae Figure 1. Intraocular pressure and examination of the fundus, including B-scan ultrasonography, were within normal limits. The patient underwent a lensectomy and anterior vitrectomy in his left eye. At six months postoperatively, the BCVA was 20/200.

## 3. Discussion

Anterior megalophthalmos is an uncommon hereditary condition characterized by bilateral megalocornea and an enlargement of iris–lens complex [4]. In anterior megalophthalmos, megalocornea is accompanied by retroposition of the iridolenticular diaphragm with a deepened anterior chamber, enlarged ciliary ring with zonular abnormalities and iridodonesis, lens enlargement, subluxation, and, frequently, premature cataract formation. The axial length of the vitreous chamber is thus reduced but the overall axial length of the globe is usually normal [5, 6].

Lens dislocation is frequently observed secondary to ocular trauma. It is the most common cause of lens luxation-subluxation, accounting for about 53% of all cases [7]. Blunt ocular trauma compresses the eye in the anterior-posterior direction expanding the eye and stretching the zonules [8]. The direction of dislocation determines management. Anterior lens dislocation requires lens removal. Refractory glaucoma, persistent uveitis, and corneal damage are indications for urgent or emergent intervention. Posterior dislocation may be managed conservatively by correction with aphakic contact lenses. However, in cases within posterior dislocation, lens removal should be considered in patients that do not tolerate correction, are symptomatic despite correction, or develop glaucoma or persistent uveitis. If the patient undergoes surgery for another posterior chamber pathology lens removal may also be considered [9]. Less common etiologies of lens dislocation include spontaneous dislocation, intraocular tumors, and connective tissue disorders such as Marfan syndrome and Ehlers-Danlos syndrome [7]. In anterior dislocations of the lens, the treatment is urgent surgical removal of the lens to avoid severe complications such as pupillary block glaucoma, corneal edema, ocular hypertonia, retinal detachment, and permanent corneal decompensation from prolonged contact of the lens with the corneal endothelium [10].

Our patient underwent successful surgical extraction of the dislocated lens and vitrectomy with a plan for future intraocular lens implantation.

In conclusion, anterior megalophthalmos is an uncommon congenital condition, and comprehensive management of complications is necessary for optimal visual outcomes and long-term satisfactory results.
